# “Vision for Action” in Young Children Aligning Multi-Featured Objects: Development and Comparison with Nonhuman Primates

**DOI:** 10.1371/journal.pone.0140033

**Published:** 2015-10-06

**Authors:** Dorothy Munkenbeck Fragaszy, Hika Kuroshima, Brian W. Stone

**Affiliations:** 1 Psychology Department, University of Georgia, Athens, Georgia, United States of America; 2 Psychology Department, Kyoto University, Kyoto, Japan; Università di Parma, ITALY

## Abstract

Effective vision for action and effective management of concurrent spatial relations underlie skillful manipulation of objects, including hand tools, in humans. Children’s performance in object insertion tasks (fitting tasks) provides one index of the striking changes in the development of vision for action in early life. Fitting tasks also tap children’s ability to work with more than one feature of an object concurrently. We examine young children’s performance on fitting tasks in two and three dimensions and compare their performance with the previously reported performance of adult individuals of two species of nonhuman primates on similar tasks. Two, three, and four year-old children routinely aligned a bar-shaped stick and a cross-shaped stick but had difficulty aligning a tomahawk-shaped stick to a matching cut-out. Two year-olds were especially challenged by the tomahawk. Three and four year-olds occasionally held the stick several inches above the surface, comparing the stick to the surface visually, while trying to align it. The findings suggest asynchronous development in the ability to use vision to achieve alignment and to work with two and three spatial features concurrently. Using vision to align objects precisely to other objects and managing more than one spatial relation between an object and a surface are already more elaborated in two year-old humans than in other primates. The human advantage in using hand tools derives in part from this fundamental difference in the relation between vision and action between humans and other primates.

## Introduction

An allocentric frame of reference uses the relationship between two or more objects external to the body to define the location of things in space [1–3]. When manipulating an object in relation to another object or a surface within arm’s reach, the actor uses an allocentric frame of reference, and in this case the handled object moves in relation to other relevant components of the set [4]. Managing mobile frames of reference between objects requires detecting and maintaining the relations in an allocentric frame of reference, and without regard to one’s own position. This analysis suggests why moving an object in relation to other mobile objects and in relation to substrates in a goal-directed task can be challenging. Mobile frames of reference add degrees of freedom to the system; managing multiple degrees of freedom of movement is more difficult than managing fewer degrees of freedom [5]. In the case of object manipulation, mobile frames of reference between or among objects and surfaces may be monitored and managed visually, and/or using other senses, such as the haptic sense (as when tying a knot in the dark, for example). In the human perceptual and neuropsychological literature, visual monitoring, leading to anticipatory manual action (such as shaping the hand prior to contact with an object during grasping), has been highlighted as the process most responsible for managing both egocentric and allocentric spatial frames of reference in manual action (e.g. [6]).

The neuropsychological literature regards visual processing as involving the functioning of two distinct neural systems, linked to two projection pathways in the visual cortex. The dorsal pathway, colloquially identified as the “where” or “how” system, or “vision for action” system, is thought to process spatiotemporal properties of visual events. It is thought to be sensitive to geometric properties of objects relevant to grasping and manipulation [7–9]. The ventral pathway, colloquially identified as the “what” system, supports object recognition and categorization, and is thus sensitive to stable properties of objects, such as shape. Routine actions with objects require the complementary functioning of these two systems [6, 9, 10].

How integrated functioning of the two visual systems develops in humans is not yet well understood. The two systems may develop on somewhat independent temporal trajectories, at least within the first two years of life. The object recognition system seems to function at a more mature level in the first year of life than does the vision for action system [7, 11–15]. However, both systems undergo substantive changes in the second year of life, and it appears that they become more integrated [16, 17].

Children’s performance in object insertion tasks (fitting tasks) provides one index of the striking changes in the development of vision for action in the second year of life. In these tasks, the participant is asked to insert a thin rectangular object into a narrow slot, and the key dependent variable is the angle of the long axis of the object relative to the slot. Adult patients with impaired dorsal stream function can visually discriminate between aligned and misaligned rectangles and slots but cannot correctly align the rectangle to the slot themselves [18], and children with Williams syndrome similarly have particular difficulty with this task [7].

Developmental studies indicate that normally developing children between 14 and 18 months given an object to insert into a slot did not manage this task consistently, but that children 22 months and older did so [19]. The older children adjusted the position and shape of their hand in the appropriate manner to grasp and rotate the object for insertion, indicating that vision is used in planning the action to achieve alignment prior to contact with the object [19]. Street et al. [20] examined in more detail how performance with a fitting task (using a thin disc, 8. 5 cm x 0.9 cm, and a linear slot, 10.5 cm x 1.5 cm) differed in children 18 months old vs 24 months old. This task does not require recognition of object shape, but it does require perception of geometric properties of the object (axes, size, orientation) thought to be used by the dorsal stream in visually guided action, and perception of the matching properties of the slot. In accord with Örnkloo and von Hofsten’s [19] findings, Street et al. [20] report that the 18-month old children typically did not achieve close alignment (± 5°) of the disc with the slot, but that the older children did. When the younger children were given the disc already aligned for insertion, they inserted it easily, whether the slot was oriented vertically or horizontally, as long as they did not re-orient it with respect to the slot prior to attempting to insert it. Interestingly, they found that the younger children could effectively orient their empty hands to insert them in the slot. Thus the younger children perceived the orientation of the slot, and they recognized something about the importance of orienting the body for insertion, but they could not rotate objects held in the hand to achieve the same outcome. The authors interpret their findings as indicating that the integration of object properties into planned action undergoes substantial change in the second year of life.

Street et al.’s [20] findings recall McCarty, Clifton and Collard’s [21] report that very young children oriented familiar objects (e.g., hairbrush) properly with respect to their own bodies before they were able to do so when using the same object on another person (or doll). In general, it appears that moving an object toward a stationary feature of the environment is inherently more difficult for children than moving a part of their bodies with respect to the same stationary feature of the environment, or moving an object to their bodies. In the terminology of spatial frames of reference, working within an allocentric frame of reference between two objects is more challenging for action than working within an egocentric frame of reference.

Young children demonstrate a dissociation between selecting objects to use as tools and effectively orienting objects while using them as tools; they are more effective at the former than the latter (e.g. [22]). The perspective adopted in this report suggests that children’s limitations at two to three years of age in using tools do not stem from insensitivity to the properties of objects they might use, or what they need to accomplish with a given tool. Rather, we suggest that, as in the fitting task, young children cannot effectively manage the allocentric relations present in many tool-using problems, and particularly, they cannot manage these relations visually–the limitation can be seen as a limitation in “vision for action”.

Lockman [4] argues that developmental changes in the management of allocentric spatial relations supports the elaboration of tool use in young children. This notion is in accord with the idea that developmental changes in the “vision for action” system in young children proceed first from supporting alignment of the body with respect to an environmental feature, such as a slot (an egocentric relation), to later supporting alignment of an object to an environmental feature (an allocentric relation). Lockman’s argument falls within the theory of ecological psychology now identified as Perception-Action theory. A key tenet of Perception-Action theory is that individuals generate behavior to learn about current circumstances; they use action to generate perceptions, and they use perception to guide actions. Hence, exploratory behavior is viewed as critically important for learning how to achieve a goal through action. Individuals learn about the affordances of the materials and of their actions with the materials through exploration [23]. From the perspective of Perception-Action theory, when faced with an insertion or alignment problem involving allocentric spatial relations that exceed their current ability to manage visually, young children should act in ways to discover the relevant relations. Adult humans can detect a variety of affordances of objects for use as tools from acting on the objects without vision [24]. Klatzky et al. [25] show that by four years of age, children can accurately select the most appropriate object for use as a tool in a given task in part through haptic exploratory procedures (touch without vision). In this study, we consider if children adopt haptic strategies to achieve alignment of one object to another object, and what kinds of problems elicit these actions. This analysis is relevant to understanding the process by which young children explore allocentric relations.

The consensual view is that relating two spatial elements to one another in an integrated manner (i.e., concurrently) is more challenging than relating two elements sequentially, and we expect to see this pattern in children’s placement of objects relative to a fixed environmental feature, as in an insertion task or alignment problem, as well as in a tool-using problem. The bulk of the neuropsychological literature about adults managing allocentric spatial relations in manipulation problems focuses on visual perception and the achievement of anticipatory positioning of the hand to the object and of one object to another. The developmental literature reflects the same emphasis, with greater attention to the emergence of anticipatory visual processing (e.g, [20]). Nevertheless, haptic perception through active touch is fundamental to normal human manual function, providing a wealth of information about the relationships of objects to one another (e.g., slip, penetration, relative mass, etc.; [26]. In addition to insertion tasks such as those used by Street et al. [20] and Örnkloos and von Hofsten [19], many object manipulation tasks presented to young children provide haptic as well as visual feedback to the children about objects’ spatial relationships to one another (e.g., seriating cups of graduated sizes, stacking blocks, using a brush on a doll’s hair [21, 27, 28]). However, findings from these studies do not directly address the role of haptic information in children’s efforts to complete the problems.

This study provides an initial examination of children’s use of haptic information to align an object. The alignment task permitted haptic feedback about correct alignment when presented in the three-dimensional format (inserting an object in a matching cut-out in an otherwise uniform surface) but lacked haptic feedback when presented in the two-dimensional format (placing the object on a drawing with contours matching the object’s contours). In this way, reliance on the use of vision for action (evident in anticipatory actions) could be dissociated from reliance on exploratory haptic actions as the basis for placement. We expected that younger children would show a measurable difference in performance between the conditions that do and do not afford haptic information, indicating that they depended in part upon haptic information to align the object to the surface. Older children should be more likely to rely on visual perception alone to manage the same problem, and thus perform equivalently in the two versions of the task.

We also investigated how young children arrange objects of different shapes in relation to a substrate. The study is designed to evaluate the hypothesis from Fragaszy and Cummins-Sebree’s [29] model of spatial reasoning that increasing the number of allocentric spatial relations (embodied in objects with asymmetrical features) to be managed concurrently increases the challenge of a manipulation problem. The task we presented is similar to the fitting task used in previous studies in that the child was asked to move one object into alignment with a feature of a spatially fixed surface. However, rather than insert an object through an aperture, as in the previous studies, in this study (as in Shutts et al.’s work [30]) the child was asked to place an object in a matching cut-out in a tray or on a matching contour (drawing) of the object on a flat mat. The objects we presented varied systematically in the number of features (from 1 to 3) that had to be aligned concurrently for the object to fit the matching cut-out or contour. Inserting an object with a symmetrical outer contour (e.g., a bar) requires managing one relation (one feature) between object and the cut-out; aligning a single edge serves to align the entire object. The fitting task used by Street et al. [20] is an example of this kind of problem. In contrast, aligning an asymmetrical object efficiently requires managing at least two features concurrently. For example, efficiently aligning a cross with the cross arm set off-center into a cross-shaped cut-out requires simultaneously managing the two planar axes of the object with respect to the cut-out. If the actor deals with the two axes sequentially, initially aligning the long axis of the stick with the long axis of the cut-out, for example, the cross piece would be at the wrong end of the long feature (i.e., the cross stick would be upside down with respect to the cut-out) half the time, on average. Each additional asymmetrical feature increases the number of relations that must be managed concurrently to align the object correctly, and decreases the probability of success that would result from using a sequential strategy. In accord with the hypothesis that an increasing number of concurrent relations increases the challenge of a problem, we predicted that (1) the number of asymmetrical components in the object to be inserted would directionally affect the number of insertion attempts per trial and (2) that children would align the asymmetrical features of the objects less consistently than the symmetrical feature (the long axis of the stick).

In accord with the hypothesis that individuals generate exploratory behavior to detect relational affordances, we predicted (3) that as the number of relations increased the children would make increasing use of exploratory actions, and (4) that younger children would make proportionally greater use than older children of actions providing haptic information. Exploratory actions could involve movements of the stick or the hand on the surface, movements combining the stick with the surface, or visual inspection of the stick and/or the surface. We were particularly interested in whether children would move the object on the surface in areas where the surface changes (i.e. the cut-out into which the object is to be inserted), an exploratory action that could provide haptic information about the fit between tray and object, in the three-dimensional version of the problem.

We also had a comparative aim in this project. Fragaszy et al. [31], in a study presenting a similar set of bar-, cross- and tomahawk-shaped objects and matching three-dimensional cut-outs as used in this study to chimpanzees and capuchin monkeys, found that individuals of both these species of nonhuman primates aligned the long axis of the bar to the matching axis of the cut-out at levels above chance (where chance = 25%), but generally on less than 50% of attempts. Most individuals aligned the cross piece to the correct end of the cut-out and oriented the tomahawk feature to the surface at chance levels (or below). The two species performed very similarly in these tasks, for example, making multiple attempts to insert the stick and usually sliding the stick across the surface before inserting it. They never oriented the object above the tray before contacting the tray with the stick. The authors were struck by the general absence of evidence for “vision for action” by these individuals with respect to the second and third features of the alignment problems presented by the cross and tomahawk shapes and their poor performance at aligning the single long feature of the bar shape. We therefore planned this study to examine how children who can manage one feature dealt with the second and third features in the cross and tomahawk shapes and to determine if they provided overt evidence of using vision for action to align the objects with a cut-out or a silhouette of the object.

## Methods

### Participants

Eleven two year-olds (4 girls, 7 boys), nine three year-olds (5 girls, 4 boys), and ten four year-olds (5 girls, 5 boys) participated in this study. Participants were tested within two weeks of their birthday. Two two year-old boys and one four year-old boy did not generate usable data. Thus the final data set included nine children from each age group, 14 girls and 13 boys total. Children were recruited in Athens, Georgia, from a list of parents who had expressed interest in participating in child development studies. All of the children exhibited normal vision and physical development and age-appropriate motor skills. The study was approved by the Institutional Review Board of the University of Georgia.

### Apparatus

**Fig 1:**
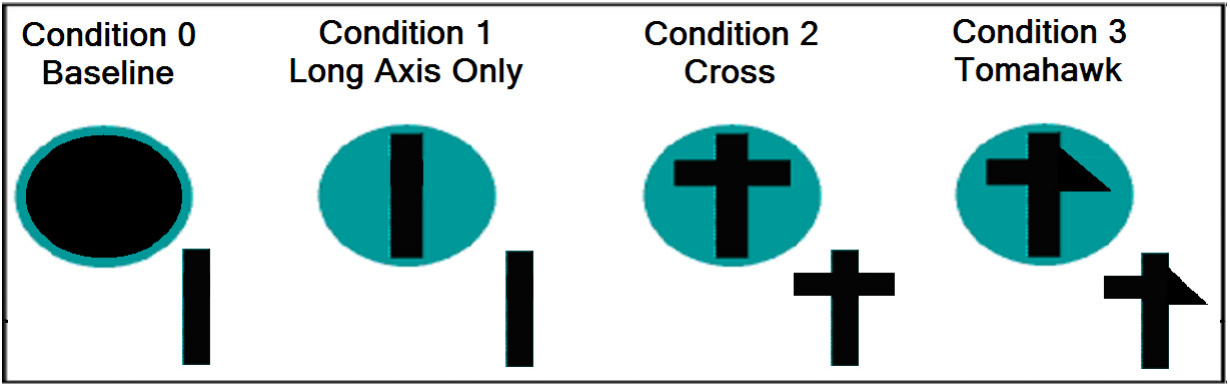
The shapes of the sticks and the matching trays for each condition. Not to scale.

For the three-dimensional task we used four different trays (Fig 1). The trays (15.3 cm in diameter X 3 cm high) were manufactured from polyvinyl chloride (hereafter, PVC), painted red except that the cut-out was left the natural gray color of the PVC. For the two-dimensional task, we used paper discs (15.3 cm diameter) reproducing in the same colors and surface dimensions as the tray the red surround and gray surface of the cut-out. Thus the discs were visually similar in color, shape and area to the trays. The discs were laminated in clear plastic.

**Fig 2:**
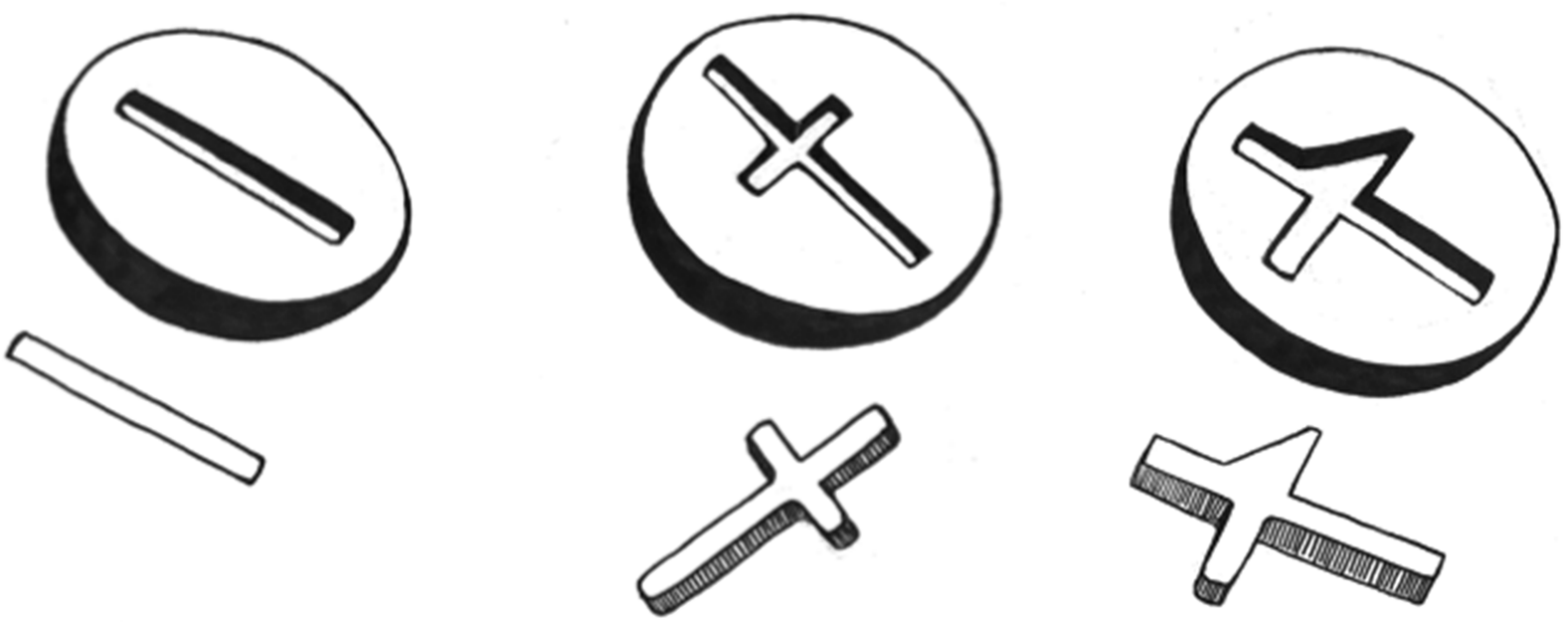
Drawings in relief of the Bar, Cross and Tomahawk sticks and the matching trays presented to two, three and four year-old children. The perpendicular arm of the tomahawk was triangular in shape on one side of the long arm. Each side of the triangle was 4.7 cm in length. The long shaft of the stick was 11.5 cm long, and 1 cm in cross-section. The Bar and Cross sticks, and the trays with cut outs matching these other shapes, were of similar dimensions.

We used the same sticks in both two and three-dimensional tasks (Fig 2). The first stick (“Bar”) was a cylindrical PVC rod 11.5 cm long X 1 cm diameter. The second stick (“Cross”) was rectangular PVC (1 cm^2^ in cross-section) with a long arm (11.5 cm) and a shorter perpendicular arm (4.8 cm) positioned 2 cm from one end of the long arm. The third stick (“Tomahawk”) was made from the same rectangular PVC, with one long arm and one perpendicular arm positioned 2 cm from one end of the long arm. The perpendicular arm of the tomahawk was triangular in shape on one side of the long arm. Each side of the triangle was 4.7 cm in length.

To familiarize the child with the three-dimensional task we first presented a gray tray (PVC) with a rimmed, solid, circular surface (12.5 cm diam) painted red and just large enough to accommodate the stick at any rotational angle. The second, third and fourth trays (for the Bar, Cross, and Tomahawk sticks, respectively) had a cut-out (1 cm deep) of the same shape and slightly wider (2 mm each side) than the matching stick (Fig 2). For the two-dimensional condition, we first presented a plain red disc of the same diameter as the tray, and then the second, third, and fourth discs with a red background and a drawing (in gray) of the Bar, Cross, or Tomahawk of the same dimensions as the exterior dimensions of the cut-out in the three-dimensional tray.

One mini-DV camera (Panasonic SDR–S100) recorded all sessions.

### Procedure

Testing was conducted at the child’s home in 2㎡ of open floor space. Two experimenters conducted each session. Following a few minutes of conversation with the parent and the child, a mat was spread on the floor in the selected area. The participant was seated on the mat next to Experimenter ‘A’. Experimenter ‘B’ operated the video camera in front of the participant and Experimenter ‘A’ at a distance of about 2 meters.

In two warm-up trials, Experimenter ‘A’ placed the solid circular tray in front of the participant and twice demonstrated deliberately placing the stick on the surface of the tray. Then she passed the stick to the participant and asked him/her to place it on the tray in the same fashion. If the participant complied, Experimenter ‘A’ presented the open disc in front of the participant, repeated the same demonstration with the disc, and then asked the participant to put the stick on the gray area of the disc. Following the child’s compliance with these requests, we proceeded to conduct the experimental trials.

In each of the three-dimensional and two-dimensional versions of the task, we presented 4 trials with the Bar and 5 trials for each other Shape (14 trials per dimension; 28 trials total). Per dimension, if the child completed the four trials with the Bar, we presented the Cross, and if he/she completed five trials with the Cross, we presented up to five trials with the Tomahawk. We adopted this order so as to allow the child to experience mastery at each level per dimension before facing the next (more difficult) level. Each test session lasted no longer than 15 minutes, and we tested each participant once. The orientation of the cut-out in the tray (or the gray outline of the stick on the disc) with respect to the participant was balanced across trials. For the Bar, the angles were 0, 45, 90, and 135 degrees. For the Cross and Tomahawk, the angles were 0, 45, 90, 180, -135, -90, and -45 degrees (so that the cross part of the cut-out was toward or away from the participant). We placed the stick on the left or right of the tray (or the disc) in a balanced order across trials. We placed the shaft of the stick in line with the cut-out and with the cross piece away from the participants.

Assignment of 2D and 3D trials alternated throughout the session, beginning with the warm-up trials. Participants were assigned to start with 2D or 3D in a balanced order. Experimenter ‘A’ placed the tray or the disc and the correct stick and asked the child to insert the stick into the cut-out in the tray or to place the stick on the gray area of the disc, to cover up the gray area. The trial ended when the participant placed the stick on the tray or the disc and removed his/her hand, or when the participant said “I’m done” or left the testing area for more than three minutes. If he/she stopped attending to the apparatus for longer than a minute before placing the stick, Experimenters verbally encouraged the participant to continue with the task. If a child lost interest in the task, Experimenter ‘A’ offered the child an opportunity to play with a “sorting box” for a few minutes, and then re-offered the tray and stick.

### Coding

We coded video with the Observer 5.0 software (Noldus Information Technology). Table 1 lists dependent variables, described below. Trials began the moment the participant picked up the stick and lasted until the task was solved or the last contact was made between stick and tray or disc. We noted if the child succeeded in inserting the stick fully into the cut-out, or placing the stick fully and exclusively over the gray area of the disc matching the shape of the stick. This constituted a “success”. We also noted per trial if the child at any time rotated the stick above the tray or disc, while apparently visually comparing the orientation of the stick to the orientation of the cut-out in the tray or the gray area of the disc. This was called an “Above tray” attempt and was coded once per trial.

**Table 1:** Dependent variables with definitions.

**SCORED PER TRIAL**	
**Success**	Stick inserted into the cut-out (three-dimensional task), or aligned with the contour (two-dimensional task)
**Above Tray Action**	Stick moved horizontally above the tray or disc before making contact, with close visual attention to the stick and the tray or disk below
**Number of Attempts**	Each attempt was a particular combination of the hand, angle of the stick with respect to the horizontal plane, alignment of each feature of the stick to the matching cut-out or contour and movement of the stick on the tray or disc. A change in any one of these features constituted the end of one attempt and the start of a new attempt. A break of ½ second or longer in contact with the tray or disc also ended an attempt.
**SCORED PER ATTEMPT**	
**Position of Shaft (Long Feature)**	Alignment to the long feature of the cut-out or contour, scored categorically in 45° octants using a clock-face rubric, where 12–6 indicated correct alignment and incorrect positions were scored as 3–9 (perpendicular), or 1–7 or 10–4 (intermediate between aligned and perpendicular octants)
**Position of Cross Feature**	Aligned (cross feature of the stick closer to the matching cross feature on the cut-out or contour) or Not aligned (closer to the end of the stick opposite the matching end)
**Position of Tomahawk Head**	Matching (the left-right orientation of the tomahawk feature on the cut-out or contour) or Not matching (reversed orientation)
**Angle**	Angle of the long axis of the stick with respect to the tray or disc upon contact: 90 if within 10° of 90°, 0 if within 10° of 0°, 45 if between these positions
**Surface Assistance**	Scored in 3D trials only. Moving the stick across the boundary between the tray’s flat surface and the cut-out

**Fig 3:**
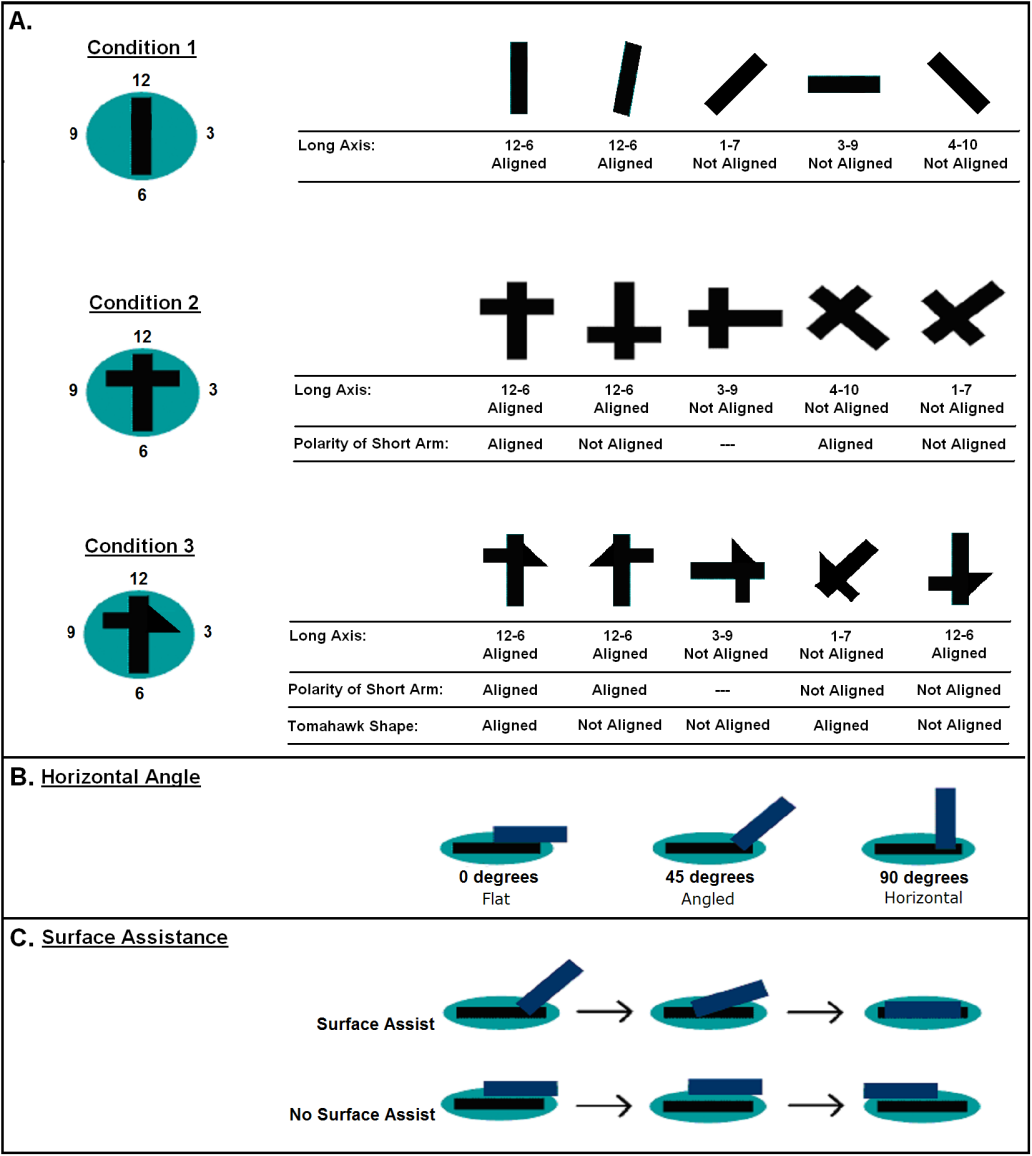
Illustrations of dependent variables for alignment of the sticks. (A) Alignment of the long and asymmetrical portions of the stick. Shown in plan view, with clock face numbers used to categorize alignment of the long axis of the stick relative to the long axis of the cut-out (defined as 12–6). (B) Angle of contact, shown in side view. (C) Surface assistance, showing temporal succession of a successful attempt where the stick placement was guided by the cut-out. Shown in side view.

All other variables were coded per discrete attempt. An attempt was operationally defined as contacting the tray with the stick. If the child struck the tray with the stick repeatedly in a bout of drumming/hammering, we scored the bout as one attempt. First we coded the alignment of the stick with respect to the cut-out in the tray (as seen from above) using a clock-face rubric (Fig 3) (i.e., perfect alignment of the Bar was indicated as 12–6 alignment). If the stick was aligned to the cut-out, or within 22.5° of the cut-out to either side, we considered it at 12–6 alignment. If the stick was perpendicular to the cut-out or within 22.5° to either side of perpendicular, we considered it 3–9 alignment. We pooled alignments in the other two quarters. For Cross and Tomahawk trials, where the stick had an asymmetrical end, we coded the alignment of the cross piece of the stick to the matching piece of the cut-out as ‘Aligned’ or ‘Not aligned’, ignoring the 3–9 case where the position of the cross piece of the stick is equidistant to the cross piece of the cut-out. For Tomahawk trials, we coded whether the left-right orientation of the head of the tomahawk stick matched that of the tray or disc.

Next, we coded the angle of the stick with respect to the horizontal plane as it touched the tray. If the stick was oriented flat against the tray or no more than 10° off horizontal, we coded it as a 0° angle. If it was within 10° of perpendicular to the tray surface, we coded it as a 90° angle (and the clock-face alignment was ignored). If it was between these two values, in the larger range from 10° to 80°, we coded it as a 45° angle. The third variable was the hand used. The final variable, coded only in 3D trials, was whether the participant moved the stick across the boundary between the tray’s flat surface and the cut-out. We called this action “surface assistance”. Examples of surface assistance are: (1) inserting the tip of the stick into the cut-out and then pivoting the other end of the stick and (2) inserting the tip of the stick into the cut-out and then sliding the tip forward while lowering the rest of the stick into place. Striking the surface with the stick as a hammer or drumstick and spinning the stick while it was fully supported on the tray were not coded as surface assistance.

A particular composite value of alignment, angle, hand, and surface assistance made up an individual attempt, and any time one of these variables changed to a new discrete value, or if the stick was removed from the surface for more than half a second, then we considered that attempt finished.

The data were coded by one person (HK). Following training with a second, experienced coder (BS) the primary coder demonstrated reliability of coding by comparing data from five infants’ data (14.8% of the total data set). The two coders' data matched on 91% of coded events (agreement divided by the sum of agreement plus disagreement) for the two variables coded per trial (success and Above tray attempts). For the four variables coded per attempt (Alignments, Angle, Surface assistance, Hand use), the two coders achieved Cohen’s K = +0.70 (Alignment) to +0.87 (Hand use). The complete data set is presented in S1 Table.

### Analysis

Our analyses examined predictions relating performance to the independent variables Age, Shape, and Dimension. We used a 3 (ages) x 2 (number of spatial dimensions) x 3 (shapes) repeated measures design, where each child received the task with three shapes in both dimensions. On average, two year-olds participated in 4.1 Bar trials (SD = 0.33), 5.0 Cross trials (SD = 0.5), and 1.4 Tomahawk trials (SD = 1.4). Three year-olds participated in 4.2 Bar trials on average (SD = 0.67), 5.0 Cross trials (SD = 0), and 5.0 Tomahawk trials (SD = 0). Four year-olds averaged 4.0 Bar trials (SD = 0), 5.0 Cross trials (SD = 0), and 4.7 Tomahawk trials (SD = 1). The data set is composed of 371 completed trials, and 1,542 attempts. Dependent variables per trial included outcome (Success or Fail), number of attempts, and presence or absence of Above tray attempts. Preliminary evaluation of the data set indicated outliers, missing cells, non-normal distribution and unequal variance across groups for number of attempts per trial and outcome per trial and therefore we used non-parametric statistical tests for all inferential analyses.

Dependent variables per attempt reported here include hand used, clock-face position of the long axis of the stick with respect to the cut-out or its outline on the disc, and for Cross and Tomahawk shapes, alignment of the cross end of the stick with respect to the matching part of the cut-out or its outline; and for Tomahawk only, the alignment of the triangle side of the tomahawk with respect to the matching part of the cut-out or its outline. We calculated the proportion of attempts in which particular categories of alignment were coded. In the 3D condition only, we also coded per attempt the occurrence or absence of surface assistance and the occurrence or absence of an Above-tray attempt. We calculated a handedness index for each child as the sum of all attempts with the right hand divided by the total number of attempts with either hand.

For the proportion of trials ending in success and in the number of attempts per trial, and for proportion of attempts in which the parts of the stick were aligned, we analyzed the effect of Shape using Friedman tests, followed by pair-wise comparisons using the Wilcoxon signed rank test for matched pairs to identify which shapes differed if a significant overall effect emerged. Alpha was adjusted to p = .017 for the post-tests (Bonferroni correction). We tested the effect of Age using Kruskal-Wallis tests for 3 samples followed by pair-wise comparisons between age groups using Wilcoxon-Mann-Whitney tests for independent samples. As before, alpha was adjusted to p = .017 for the post-tests. We tested the effect of Dimension on these variables using the Wilcoxon signed ranks test for matched pairs. For Shape and Dimension, we used 1-tailed probabilities to evaluate our directional predictions; for Age we used 2-tailed probabilities. We examined the relationship between individuals’ success at placement and number of attempts using Spearman correlations. For Wilcoxon signed ranks tests where N > 15 and for Wilcoxon- Mann-Whitney tests where n > 10 and m > 10, we report Z scores, in accord with the recommendations of Siegel & Castellan [32].

We tabulated the number of children that performed Above Tray actions, and the frequency of these events, by Age, Shape and Dimension, and analyzed the distribution of these data for age and for shape using goodness-of-fit tests, with H_0_ = equal distribution of events across age and shape. As we did not predict the occurrence of these actions, these analyses are exploratory. We examined the relation between the frequency of using Above tray movements and the proportion of successful trials over all conditions, the relation between handedness index and proportion of successful trials, and the relation between number of attempts and success using Spearman correlations.

## Results

We begin by evaluating the children’s performance in terms of percentage of successful trials and the number of attempts per trial. Children succeeded at inserting the stick in the cut-out or placing it correctly on the disc in 73% of trials (269 out of 371). They made on average 3.3 attempts to place the stick on each trial when they succeeded (Median = 2), and 6.3 attempts when they failed (Median = 3). Examples of children of different ages placing each of the sticks into the cut-out or onto the disk are shown in supplemental materials S1 Video– S7 Video. Overall, individual success at placement was uncorrelated with number of attempts (r_s_ = -0.0166, p = 0.47). The value of the handedness index (HI) per child ranged from .22 to .91 (Median = .63) and HI was uncorrelated with proportion of trials in which children succeeded at placing the stick (Spearman’s r_s_ = 0.05, N = 27). We do not consider hand use further.

**Fig 4:**
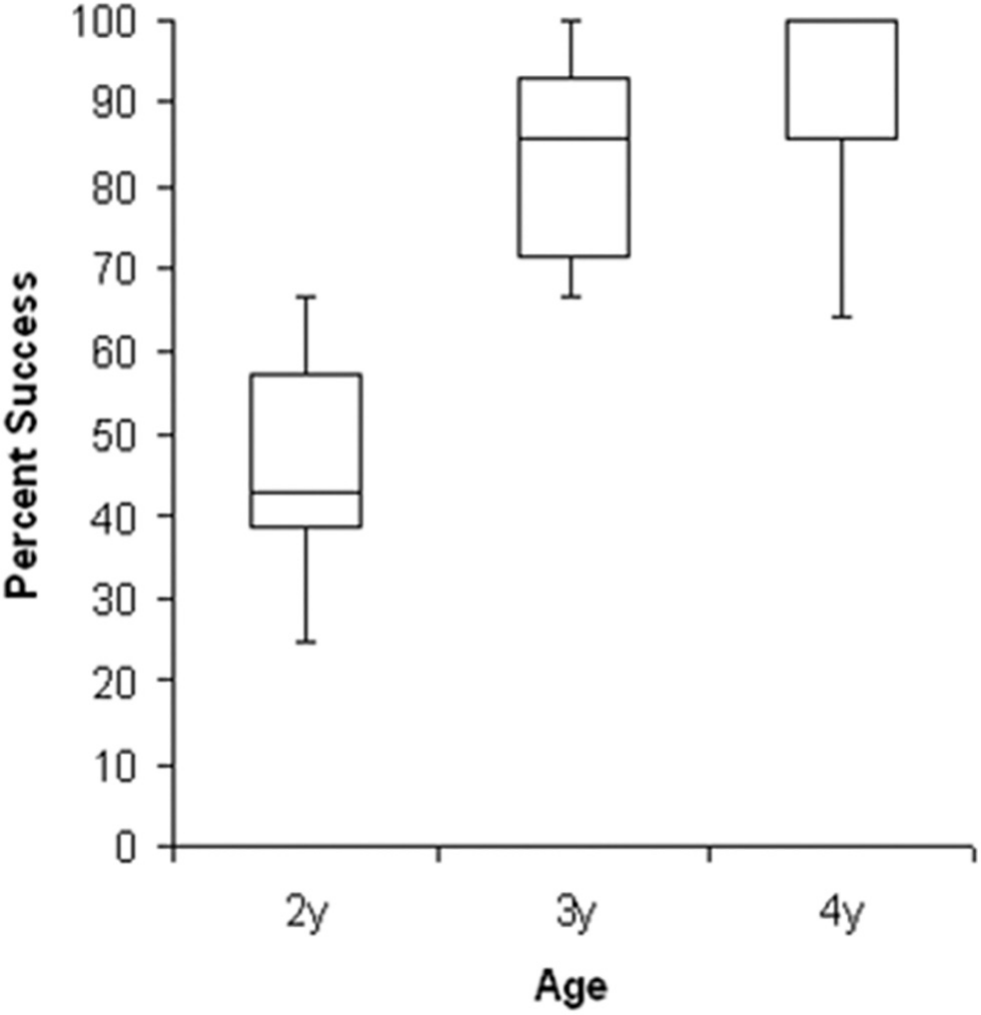
Percentage of trials in which children of each age group succeeded at placing the stick into the cut-out or onto the form on the disc. Boxes represent interquartile range (IQR), with median in the middle. Upper whiskers depict the lower of maximum data point or 1.5 x IQR. Lower whiskers depict the higher of minimum data point or 1.5 x IQR. Most extreme outlier beyond each whisker is shown by *.

Age groups differed significantly in percentage of trials in which they succeeded at placing the stick (pooled shapes) into the groove or onto the form on the disc (Kruskal-Wallis, N = 27, df = 2, corrected H = 16.84, p < 0.001). Two year-olds succeeded in 43% of trials, and three and four year-olds in 86 and 88%, respectively (Fig 4). In pair-wise comparisons, two year-olds varied significantly from three year-olds (W_x_ = 45.5, n = 9, m = 9; p <0.00001) and from four year olds (W_x_ = 45.0, n = 9, m = 9; p <0.00001). Three and four year-olds did not differ from each other (W_x_ = 76, n = 9, m = 9; p > 0.20). Boys and girls achieved equivalent success at all ages (Median = 66.5, boys, 86, girls; Wilcoxon-Mann-Whitney, m = 13, n = 14, Z = -1.29, p > 0.09) and made similar numbers of attempts (Median = 62.5, boys, and 48, girls; Wilcoxon-Mann-Whitney, m = 13, n = 14, Z = -0.49, p > 0.31) and we do not consider sex differences further. We explore the effects of Age, Shape and Dimension on performance in more detail below.

### Effects of Shape on Number of Attempts and Success

**Fig 5:**
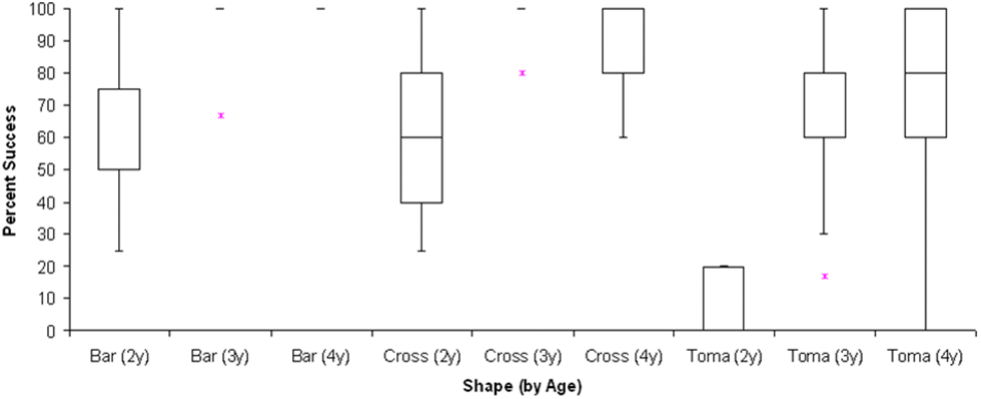
Percentage of trials in which children of each age group succeeded at placing the stick into the cut-out or onto the form on the disc, according to shape. Boxes represent interquartile range (IQR), with median in the middle. Upper whiskers depict the lower of maximum data point or 1.5 x IQR. Lower whiskers depict the higher of minimum data point or 1.5 x IQR. Most extreme outlier beyond each whisker is shown by *.

Analyses of Shape address the directional predictions that increasing the number of features to be aligned increases the difficulty of the problem, and that this effect is stronger for the younger children. Findings support this prediction. The shape of the stick affected the number of attempts the children made per trial to align the stick to the groove or the disc (Friedman, N = 27, df = 2, *X* ^2^ = 34.45, p < 0.001). Children tended to make more attempts per trial with the Cross than with the Bar (Median = 2.33, cross, vs. 1.77, bar; Wilcoxon signed ranks, N = 26, Z = -1.943, 0.017 < p < 0.026), and made significantly more attempts with the Tomahawk (Median = 8.25) than with the Cross (Wilcoxon signed ranks, N = 27, Z = 3.87, p < 0.00005) or the bar (Wilcoxon signed ranks, N = 27, Z = 3.87, p < 0.00005). Shape also affected children’s success at placing the stick correctly (Friedman, N = 27, df = 2, *X* ^2^ = 29.04, p < 0.001; Fig 5 illustrates success with different shapes at each age). They succeeded at placing the Bar in 86% of trials, and the Cross in 84% of trials, but the Tomahawk only in 49% of trials (Median values). Wilcoxon signed ranks tests revealed that children succeeded equally often with Bar and Cross (N = 13, T^+^ = 61.5, p > 0.15; 14 children had tied scores), but significantly less often with the Tomahawk than with the Bar (N = 23, Z = -4.17, p < 0.00003) or the Cross (N = 22, Z = -4.07, p <0.00003). The Tomahawk condition challenged all three age groups, but especially the two year-olds. Two year-olds succeeded on a lower percentage of trials (Median = 0) with the Tomahawk than did three and four year-olds (pooled) (Median = 80%) (Wilcoxon-Mann-Whitney, n = 9, m = 18; Z = -4.89, p < 0.00003). Three and four year-olds did not differ from each other on the percentage of trials with the Tomahawk that were successful (Wilcoxon-Mann-Whitney, n = 9, m = 9; W_x_ = 82.5, p > 0.39).

### Effects of Dimension on Number of Attempts and Success

Analyses of Dimension address the directional predictions that presence of haptic information about the position of the stick with respect to the groove (present in the 3D version of the task) decreases the difficulty of the problem, and that this effect is greater for younger children. Findings support these two predictions. Dimension significantly affected the number of attempts children made to align the sticks. On 2D trials, children made 2.0 attempts per trial, and on 3D trials they made 4.6 attempts per trial (Median values; Wilcoxon signed ranks, N = 27, Z = -4.04, p < 0.001). Dimension also significantly impacted children’s success at placing the sticks, but in the opposite direction of number of attempts. Children succeeded on 71% of 2D trials vs. 80% of 3D trials (Median values; Wilcoxon signed ranks, Z = -2.92, p = 0.003). The effect of Dimension on success was most evident for the two and three year-olds (see Table 2). Eight of nine two year-olds and seven of nine three year-olds had a greater proportion of completed (successful) trials with 3D than 2D presentations (total = 15 of 18; Χ^2^ = 8.00, df = 1, p < 0.005). The four year-olds varied essentially randomly on this variable, with three succeeding more often with 3D tasks, four performing equivalently on both, and two performing better on the 2D. Three four year-olds succeeded on all trials in both conditions.

**Table 2:** Percent of trials completed on two and three-dimensional tasks by age groups.

DIMENSION	AGE		
	2 Years	3 Years	4 Years
**2D**	27	79	86
**3D**	62	88	89

### Effects of Shape and Dimension on Alignment of Specific Features of the Sticks

Children’s alignment of the shaft (long feature), cross feature, and the tomahawk feature did not differ significantly between two and three dimensions (Wilcoxon signed ranks, N = 27, all Z’s < |1.50|). Therefore, all analyses concerning the effect of shape were conducted with the pooled data.

Children almost always placed the shaft of the stick parallel to the long axis of the cut-out for the Bar (Median = 100%) and reliably for the Cross (Median = 88%) but less often for the Tomahawk (Median = 65%; Dimensions pooled in all cases). The values varied significantly across shapes (Friedman, N = 27, df = 2, Χ^2^ = 19.69, p < 0.001). In pair-wise comparisons, the percent of attempts in which the shaft was aligned did not differ significantly between Bar and Cross (Wilcoxon signed ranks, N = 19, Z = 1.54, p > 0.06), or between Cross and Tomahawk (Wilcoxon signed ranks, N = 27, Z = 1.89, 0.03 > p > 0.017), but did vary significantly between Bar and Tomahawk (Wilcoxon signed ranks, N = 26, Z =. 3.54, p < 0.0003).

Children in the three age groups aligned the cross piece of the Cross on an equivalent percentage of attempts (Median = 70, 78, and 65%, two, three, and four year-olds, respectively; Kruskal-Wallis, N = 27, Χ^2^ = 0.964, df = 2, p = 0.618). However, aligning the head of the Tomahawk correctly with respect to the left/right orientation of the head varied systematically across the three age groups. Two year-olds managed to align this feature correctly on 2% of attempts, three year-olds on 14% of attempts, and four years olds on 18% of attempts (Median values; Kruskal-Wallis, N = 27, Χ^2^ = 8.597, df = 2, p = 0.014). Two year-olds differed significantly from three year-olds (Wilcoxon-Mann-Whitney, n = 9, m = 9, W_x_ = 54.0, p = 0.002) and four year-olds (Wilcoxon-Mann-Whitney, n = 9, m = 9, W_x_ = 58.5, p < 0.009); three and four year-olds did not differ (Wilcoxon-Mann-Whitney, n = 9, m = 9, W_x_ = 89; p > 0.60) on this measure.

### Actions with the Stick On and Above the Tray or Disc

Individual children held the stick so that it was horizontal (0–10°) when it contacted the tray on 80% to 100% of trials, and there was no consistent variation in this measure across age groups or stick shapes. Children moved the stick across the surface of the tray and brought some part of the stick into contact with the cut-out (hereafter, termed “surface assistance”) on 50% of attempts with the Bar, 70% of attempts with the Cross, and 46% of attempts with the Tomahawk shape in the 3-dimensional condition. A Friedman’s test revealed that the distribution of surface assistance across shapes differed significantly from chance (N = 26; Χ^2^ = 10.140, p = 0.006; N = 26 in this analysis because one two year-old did not make any attempts with the Tomahawk in the 3-dimensional condition). Wilcoxon signed ranks tests indicated that children used surface assistance more often with the Cross than with the Tomahawk (N = 26, Z = -2.92, p = 0.003) but the differences between Bar and Cross and Bar and Tomahawk were not significant.

An alternate strategy used by the children to align the stick involved moving the stick in the air, above the tray or disc, in a predominantly horizontal position and with no part of the stick in contact with the surface (termed “Above tray actions”) before contacting the tray or disc with the object. No two year-old moved the stick above the surface in this way, whereas six three year-olds did so on 10 trials total; seven four year-olds did so on 27 trials total. A goodness of fit test revealed that this distribution differs significantly from the null hypothesis that an equal number of children of each age group would move the stick above the surface at least once (X^2^ = 12.76; df = 2, p < 0.01). Children performed Above tray actions only with Cross and Tomahawk trials, and at roughly equal rates for Tomahawk (30 times out of 1031 attempts; 2.9%) and Cross (7 times out of 315 attempts; 2.2%). Dimensionality did not affect the likelihood of a child performing an Above tray action: Such actions occurred 21 times in 2D trials and 16 times in 3D trials (goodness of fit test, where H_0_ is equal distribution of Above tray actions, X^2^ < 1.0, df = 1; NS). However, those children who performed one or more Above tray actions succeeded on a higher percentage of trials than children who did not (Median = 86% vs. 61%, respectively, Wilcoxon-Mann-Whitney, N = 27, Z = -3.151, p < 0.002) and the frequency of using Above tray actions correlated positively with the proportion of successful trials over all conditions (Spearman’s r_s_ = +0.59, n = 27, p = 0.001).

## Discussion

We evaluated two to four year-old children’s ability to align an object to another object (a stick to a tray or a mat) in a fitting task. The findings provide insights into the development of vision for action [6]. They also provide support for models of spatial reasoning drawn from Perception-Action theory [4, 29], and for the proposal that children use haptic information in fitting tasks, but no support for the proposal that children use overt exploratory actions to gain haptic information. Instead, children adopted behaviors supporting visual exploration of alignment prior to placing the sticks on the surfaces, and these behaviors were positively associated with success at placing the sticks.

### The Influence of Shape

The sticks that the children placed into the tray or onto the mat contained one, two or three orthogonal features in one plane (Bar, Cross, Tomahawk shapes, respectively). To place the stick correctly, the child had to align the orthogonal features of the object with the matching cut-out or drawing. When there was more than one feature to align, the features could be aligned sequentially (which would require successive actions) or concurrently. According to Perception-Action views [29], managing concurrent spatial relations between objects is more challenging than managing single relations, leading to the prediction that the difficulty of alignment increases with each additional feature in the stick. The findings support this prediction: the challenge of the task varied in an orderly way with the shape of the objects. Children had little problem fitting the Bar or Cross compared to the Tomahawk. Children usually succeeded at aligning the Bar or the Cross to the cutout or contour (86% of Bar trials, and 84% of Cross trials), but they did so with the Tomahawk on less than half (49%) of their trials with this object. The number of attempts per trial that the children used to align the sticks followed the number of features to be aligned concurrently: children used significantly more attempts to place the Tomahawk than the Cross or the Bar, which did not differ significantly from each other. The values for the number of attempts per trial to align the Bar match well with mean values reported by Street et al. [20] for 18 and 24 month-olds inserting a disc into a slot, a task similarly involving aligning one planar feature. The similarity gives confidence that the task of aligning the Bar was approximately equal in difficulty for two year olds as inserting a disc into a slot, thus supporting comparisons of our findings for two year old children with Street et al.’s [20] findings. Our findings indicate that aligning three features concurrently is essentially beyond two year-olds’ ability, and is still a formidable challenge for three and four year-olds. This aspect of alignment develops slowly, in comparison to the alignment of one and two features, mastery of which was evident in all three age groups.

In sum, our explanation for the pattern of our findings that young children faced significantly more difficulty aligning the Cross than the Bar, and the Tomahawk than the Cross, is that in each case the additional feature present in the former compared to the latter required concurrent monitoring and control of one additional spatial relation in the form of a requirement to align an additional feature to a surface. The Bar presented a single planar feature to align; the Cross presented two, rather than one; the Tomahawk presented three.

Children of the three age groups varied significantly in their success at aligning the head portion of the Tomahawk, which varied asymmetrically around the long axis of the stick (that is, the left side differed from the right). The difficulty of aligning the head of the Tomahawk for all the children is reflected in a greater number of attempts to align this object than the others, as well as in the rate of success. Two year-olds managed to align this feature correctly on a significantly lower proportion of attempts (2%) than three and four year-olds (14% and 18% of attempts, respectively). It might seem puzzling that the children persisted at attempting to solve a problem at which they often did not succeed. However, this finding reminds us that children are motivated to solve challenging movement problems, as for example when learning to walk. Children take thousands of steps and fall hundreds of time per day while learning to walk [33].

We note that our design confounds shape with order of presentation, as we presented the sticks to all the children in a fixed order: Bar, then Cross, then Tomahawk. We did this deliberately so that children would not lose interest in the task when it was very difficult for them, as we anticipated would be the case with the objects presenting more than one feature to align concurrently. Thus it is possible that fatigue contributed to the children’s poorer performance with the tomahawk than the other two shapes. However, we saw no evidence of fatigue in the children’s performance. For example, the number of attempts increased with each new shape presented (from Bar to Cross, and Cross to Tomahawk). Moreover, the sharp difference in the proportion of trials completed with the cross vs. tomahawk shapes that was evident in all the two year-old participants indicates that shape played a larger role than fatigue, because we expect fatigue would produce a gradual rather than step-like shift in performance.

The long axis of an object (the axis of elongation) develops as a key frame of reference for object perception and object action in the second year of life [34]. In Smith et al.’s study [34], in the second experiment, when inserting objects downward into a rectangular cut-out [34 Experiment 2], two year-olds appeared to attend to the long axis regardless of whether they were inserting a simple object or a complex object. In Smith et al.’s study [34], the task required aligning only the long axis. It may be that two year-olds work first with the axis of elongation for insertion problems; however, our data suggest that by two years of age, children can successfully align a second, shorter axis as well as an object with one axis. In Smith et al.’s study [34], two year-olds took a maximum of three adjustments to align the long axis alone; two year-old participants in our study averaged less than three attempts to align the Bar shape and less than 5 to align the Cross shape and they were equally successful at aligning both objects.

However, with the Tomahawk, two year-olds were less likely than three and four year-olds to align the third axis (the asymmetric head of the tomahawk) on any given attempt, and two year-olds completed the task less often with the Tomahawk than with the Bar or Cross shapes. The second axis in our study (the cross feature) may be easier to align than the third precisely because children tended to make attempts with the object nearly flat when inserting it into a cut-out or onto a flat contour. In this situation, rotating the object clockwise or counter-clockwise (which is sufficient for aligning the first two axes, and thus succeeding with the Cross as well as the Bar) keeps it in planar view, whereas the 'flip' required to align the third axis for the Tomahawk requires temporarily rotating the object out of planar view. James et al. [35] show that 12 to 36 month-olds tend to prefer planar views when examining objects.

### The Influence of Dimension

Children succeeded at aligning the objects on 71% of 2D trials vs. 80% of 3D trials (Median values). Although this difference was statistically significant, it was not as large as one might expect if children had used haptic information effectively to explore the surface of the three-dimensional tray. Despite their higher rates of success on the 3D condition than the 2D condition, children aligned the various features of the objects to the cut-out or silhouette on equivalent proportions of attempts in the 2D and 3D conditions and typically placed the stick flat against the surface in both conditions. Moreover, the children made more attempts per trial to align the 3D objects than the 2D objects, again suggesting that their efforts to align the parts of the object to the surface were not better organized when they placed the stick into a cutout than when they placed it on a visible contour. These findings suggest that children did not act differently with the sticks in the 2D vs. 3D conditions, but that the presence of the cut-out aided the children to detect (probably haptically) when the stick encountered the cut-out. Perhaps haptic information available in the 3D condition supported more effective alignment in an incidental manner, by allowing children to detect where the stick encountered an irregularity in the surface of the tray.

As for the effect of Shape, the effect of Dimension on success was most evident for the two and three year-olds. Eight of nine two year-olds and seven of nine three year-olds had a greater proportion of successful trials with 3D than 2D presentations. The four year-olds varied essentially randomly on this variable (and they were generally successful with all the shapes in both dimensions).

Some three and four year-old children, but not two year-olds, sometimes moved the stick above the tray while looking carefully at the tray and the stick (termed “above tray movements”). Children that made above-tray movements succeeded on a higher percentage of trials than children who did not, and the frequency of such movements correlated positively with the proportion of successful trials. It appeared that the older children were aware, at least sometimes, that they could match the positions of the object and the cut-out by fine lateral movements of the stick. However, they performed these actions equivalently with the Cross and Tomahawk shapes, suggesting the strategy was not a product of perceived challenge of the task, because they were much more successful at aligning the Cross than the Tomahawk. If the Tomahawk was held in the wrong way, it had to be rotated about its shaft to correct its position. Thus this problem could not be corrected by moving the stick laterally above the tray, rotating it in a horizontal plane. Moving the stick above the tray indicates growing sophistication of vision for action but it is not tightly linked developmentally with aligning through rotation of an object about a plane, as when turning an object over from front to back.

### Development of Vision for Action

Street et al. [20] suggest that abilities to align objects through visually guided action improve dramatically in the period from 18 to 24 months; our analyses suggest that vision for action in the service of object alignment progresses between two and three years of age from aligning single features to aligning two features, and greater facility at using vision in the absence of haptic feedback concerning fit. Shutts et al. [30] found that children aged 25–30 months succeeded at placing a ball or a cube into a matching aperture or onto a matching silhouette on about 80% of trials, and unlike our findings, the children achieved equivalent success in the two-dimensional and three-dimensional versions of these tasks. In our study, both two and three year-old children succeeded on a greater percentage of 3D trials than 2D trials, and this was most evident for two year-olds (see Table 2). Jung et al. [36] took a complementary perspective, studying the process by which young children (16–33 months old) integrate rotation and translation of an object while completing a fitting task. These authors show that children between two and three years-old, compared to children16–20 months, better integrate translational and rotational components of movement while bringing a rod to a matching cut-out placed on a table in front of them. The children all attended visually to the cut-out as they transported the rod, and they all succeeded eventually at inserting the rod into the cut-out, but the older children simplified the task by keeping the rod parallel to the surface of the table, so that they had less work to do to align the long axis of the rod with the long axis of the cut-out, and they prospectively aligned the rod with cut-out, so that they needed only to rotate it slightly in a horizontal plane to insert it. Both of these latter findings are in accord with the findings from this study, in which the youngest age group (two year-olds) routinely aligned the long axis of the sticks within a few degrees of the cut-out or contour and contacted the tray or disc with the stick in a horizontal position. Putting these findings together, it appears that children by two years of age typically have sufficient visuomotor coordination to move a held object into alignment with a surface feature, managing both translation and rotation in a horizontal plane. The third year of life is an important transition period for the development of competence at aligning objects with multiple features with respect to features of surfaces and for employment of rotation about a plane (as when turning an object over from ‘back’ to ‘front’). This is particularly the case for situations providing limited haptic information relevant to placement (as when placing an object on a two-dimensional silhouette). The latter situations require placement using vision. By four years of age, children are practicing anticipatory visually-guided actions that support alignment in advance of contact of objects to surfaces, they occasionally match an object to a cut-out by turning the object over, and they can align objects equally well in 2D and 3D formats.

### Comparison with nonhuman primates

In a parallel study to the one reported here, Fragaszy et al. [31] presented Bar-, Cross-, and Tomahawk-shaped sticks and matching cut-outs to chimpanzees (*Pan troglodytes*) and tufted capuchins (*Sapajus* spp.). The nonhuman subjects were markedly less proficient at aligning the sticks with cut-outs than the children that participated in the current study. Chimpanzees aligned the long shaft of the sticks to within 22.5° the matching cut-out on 35% of their attempts, and capuchins on 42% of their attempts, vs. median values of 100%, 88%, and 66% for children in this study with the Bar, Cross and Tomahawk shapes, respectively. There was no overlap in the distributions of alignment performance by children with performance by nonhuman primates. La Cour et al. [37] presented to tufted capuchins and chimpanzees a three-dimensional placement task with Bar and Cross sticks of equal dimensions, which could have made the task easier. The nonhuman primates succeeded at aligning these sticks to cut-outs on a similar proportion of attempts as the original sticks presented by Fragaszy et al. [31], and again, with no overlap in the distribution with children. Thus it is clear that alignment of just one feature to a three-dimensional matching cut-out is challenging for nonhuman primates, even for those species that spontaneously use tools in the wild (reviewed in Sanz et al. [38] and Shumaker et al. [39]) and that are regarded as possessing well-developed manual dexterity among primates [40, 41].

The difference between humans and nonhuman primates is equally stark with respect to working with two allocentric relations concurrently. Chimpanzees and tufted capuchins in Fragaszy et al.’s [31] and in La Cour et al.’s [37] study made nearly four times as many attempts to insert the Cross stick into the cut-out compared to the Bar stick. In Fragaszy et al.’s [31] study, no individual of either species aligned the cross feature of the stick with the cross piece of the cut-out more often than expected by chance (average for chimpanzees, 46% of attempts; average for capuchins, 47% of attempts). In the Cross condition, one chimpanzee and two capuchins aligned the long axis of the stick but in the same attempt mis-aligned the cross piece of the stick more often than expected by chance, and no individual jointly aligned both features more often than expected by chance. With the Tomahawk stick, one capuchin correctly aligned the long axis, the cross piece, and the left-right orientation of the tomahawk feature more than expected by chance, and two chimpanzees aligned these three features less often than expected by chance. In short, when the task embodied managing two or more concurrent spatial relations, the nonhuman primates worked systematically only with the most familiar one (the long axis of the stick), which may also have been the most salient one for them. In stark contrast, children concurrently aligned the long segment and the cross piece of the Cross stick on about 70% of their attempts on average with no significant variation across age groups. Once again there is no overlap in the distribution between humans and nonhuman primates.

In accord with lesser success at aligning features of the sticks to cut-outs, chimpanzees and capuchins used more attempts to complete each trial than the children. Chimpanzees averaged 4.2 and 17.6 attempts per trial with the Bar stick and Cross stick, respectively; capuchins, 9.4 and 31.9 for the same conditions. Two year-old children in this study made half as many attempts to place the Bar in the cutout as the Cross (2.5 vs. 5) but were still successful in far fewer attempts than the nonhuman subjects in both conditions, and the two older age groups placed both the Bar and the Cross in less than two attempts, on average. There is no overlap in the distributions between human and nonhuman participants in any condition for the number of attempts used to place the stick into the tray correctly. This variable could also reflect persistence, as the nonhuman subjects were apt to continue to try to place the stick into the tray whereas young children, after some number of attempts, were apt to abandon the task or begin to do other actions with the stick (e.g., hammer the stick on the tray). Thus the findings for proportion of attempts that were aligned are more telling with respect to how participants managed the problem, in our view, than the number of attempts used to place the stick.

The only feature of performance where any overlap between human and nonhuman participants was evident is success at aligning the left/right orientation of the head of the Tomahawk stick with respect to the matching feature of the cut-out. As mentioned above, two year-olds managed to align this feature correctly on 2% of attempts, three year-olds on 14% of attempts, and four years olds on 18% of attempts. Three chimpanzees aligned the Tomahawk feature correctly on 8% of attempts, and four capuchins on 11% of attempts (one capuchin and one chimpanzee did not attempt to place the Tomahawk shape). Thus the human advantage in performing this part of the task emerges after two years of age.

Working with two or more allocentric spatial relations concurrently, as in stone knapping, and aligning objects precisely, as when sharpening a blade in a groove, are prerequisites for human-typical ways of making and using hand tools. Our findings suggest that managing allocentric spatial relations and managing alignments of objects to surfaces appear in early childhood, initially for one and two concurrent relations in the first two years of life, and in the next two years, for three concurrent relations. These features of manual activity distinguish us from other primates and likely support the great elaboration of tool use and other dexterous activities in our species compared to other primates, and the flowering in early childhood of human-typical forms of using hand tools.

## Supporting Information

S1 Table Data set, children’s behavior with sticks as they inserted or aligned them. Full data set analyzed in this report. A noted at the top of each column explains the meaning of the column heading and the values in the cells of that column.
(XLSX)

S1 Video Two year-old child inserting a straight stick (“bar”) into a matching cut-out. (MPG)

S2 Video Two year-old child placing a cross-shaped stick onto a matching contour. (MPG)

S3 Video Three year-old child inserting a straight stick (“bar”) into a matching cut-out. (MPG)

S4 Video Three year-old child inserting a cross-shaped stick into a matching cut-out. (MPG)

S5 Video Three year-old child placing a tomahawk-shaped stick onto a matching contour. (MPG)

S6 Video Four year-old child inserting a cross-shaped stick into a matching cut-out. (MPG)

S7 Video Four year-old child inserting a tomahawk-shaped stick into a matching cut-out. (MPG)
